# Mitochondrial ROS activates ERK/autophagy pathway as a protected mechanism against deoxypodophyllotoxin-induced apoptosis

**DOI:** 10.18632/oncotarget.22875

**Published:** 2017-12-04

**Authors:** Sang-Hun Kim, Kwang-Youn Kim, Sul-Gi Park, Sun-Nyoung Yu, Young-Wook Kim, Hyo-Won Nam, Hyun-Hee An, Young-Woo Kim, Soon-Cheol Ahn

**Affiliations:** ^1^ Department of Microbiology & Immunology, Pusan National University School of Medicine, Yangsan 50612, Republic of Korea; ^2^ Section of Pulmonary, Critical Care and Sleep Medicine, Department of Internal Medicine, Yale University School of Medicine, New Haven, Connecticut 06510, USA; ^3^ Korean Medicine Application Center, Korea Institute of Oriental Medicine, Daegu 41062, Republic of Korea; ^4^ Department of Herbal Formula, Medical Research Center (MRC-GHF), College of Oriental Medicine, Daegu Haany University, Gyeongsan 38610, Republic of Korea; ^5^ Immunoregulatory Therapeutics Group in Brain Busan 21 Project, Pusan National University, Yangsan 50612, Republic of Korea

**Keywords:** Deoxypodophyllotoxin, apoptosis, autophagy, mitochondrial ROS, ERK

## Abstract

Deoxypodophyllotoxin (DPT) is a naturally occurring flavolignan isolated from *Anthriscus sylvestris*. Recently, it has been reported that DPT inhibits tubulin polymerization and induces G2/M cell cycle arrest followed by apoptosis through multiple cellular processes. Despite these findings, details regarding the cellular and molecular mechanisms underlying the DPT-mediated cell death have been poorly understood. To define a mechanism of DPT-mediated cell death response, we examined whether DPT activates signaling pathways for autophagy and apoptosis. We demonstrated that DPT inhibited cell viability and induced apoptosis in prostate cancer cell lines, as evidenced by a mitochondrial membrane potential and expression of apoptosis-related proteins. Reactive oxygen species (ROS), primarily generated from the mitochondria, play an important role in various cellular responses, such as apoptosis and autophagy. DPT significantly triggered mitochondrial ROS, which were detected by MitoSOX, a selective fluorescent dye of mitochondria-derived ROS. Furthermore, DPT induced autophagy through an up-regulation of autophagic biomarkers, including a conversion of microtubule-associated protein 1 light chain 3 - I (LC3-I) into LC3-II and a formation of acidic vesicular organelles. Moreover, mitochondrial ROS promoted AKT-independent autophagy and ERK signaling. The inhibition of autophagy with 3-methyladenine or LC3 knockdown enhanced DPT-induced apoptosis, suggesting that an autophagy plays a protective role in cell survival against apoptotic prostate cancer cells. Additionally, the results from an *in vivo* xenograft model confirmed that DPT inhibited tumor growth by regulating the apoptosis- and autophagy-related proteins.

## INTRODUCTION

Apoptosis and autophagy are the two fundamental types of programmed cell death [1]. Autophagy is a self-degradative catabolic process in which damaged organelles and long-lived proteins are degraded and recycled to maintain normal cellular homeostasis [2]. Protein degradation occurs through via the formation of autophagosomes with a double membrane vesicle that sequesters a part of the cytoplasm. A fusion between autophagosomes and lysosomes generates autolysosomes, in which its luminal content are degraded by acidic lysosomal hydrolases [3]. The formation of autophagosomes is initiated by an induction of various autophagy genes, including microtubule-associated protein 1 light chain 3 (LC3), phosphatidylinositide 3 kinase (PI3K), Beclin-1, and autophagy genes (ATG) [4]. Although the role of autophagic cell death remains to be controversial in cancer [5], autophagy plays a cytoprotective role for cell survival against apoptosis during chemotherapy treatment [6, 7]. A recent report suggested that the suppression of autophagy with ATG7 depletion inhibits this cytoprotective action of rapamycin in the human cells, *in vitro* [8]. Meanwhile, some studies have shown that the inhibition of autophagy reduces apoptosis, suggesting that autophagy participates in the apoptosis enhancement [9]. The cytotoxic effect of autophagy could be explained by the direct self-destructive potential of massive autophagy (type II cell death). Furthermore, the role of autophagy may depend on the agents, type of cancer, stage of tumorigenesis, and status of apoptosis in cancer cells.

The PI3K/AKT/mTOR pathway, which is activated in cancer, is an important factor in autophagy [10]. The PI3K/AKT pathway is a major upstream modulator of mTORC1, which leads to the suppression of autophagy and involves cell survival and apoptosis inhibition in different types of cell [11]. Although it is well known that mTOR was activated by the PI3K/AKT pathway, it is also activated by AKT-independent regulation, such as mitogen-responsive, energy-sensing, hypoxia and amino acids [12]. In addition, several rugs such as lithium, carbamazepine and valproic acid, reduce intracellular inositol and inositol 1,4,5-trisphosphate (IP_3_) levels, subsequently inducing autophagy independent of mTOR activity [13]. Nonetheless, recent studies have demonstrated that pharmacologic inhibition of PI3K/AKT/mTOR signaling with rapamycin and its analogues is a potent cancer-selective therapeutic strategy for many tumor types. In chemotherapy, it has been reported that a signaling pathway of mitogen-activated protein kinases (MAPKs) induces autophagy in various cancer cells [14]. In particularly, an activation of extracellular signal-regulated kinase (ERK) signaling has been involved in autophagy induction by several stimuli including amino acid deprivation, aurintricarboxylic acid, β-group soyasaponins and curcumin [15–18].

Reactive oxygen species (ROS) are highly reactive oxygen-free radicals or non-radical molecules that are generated by many potential cellular sources such as nicotinamide adenine dinucleotide phosphate oxidases (NOX), xanthine oxidase, peroxisomes, endoplasmic reticulum (ER) and the mitochondrial electron transport systems [19]. ROS, generated primarily from the mitochondria play an important role in various cellular responses, including cell growth, differentiation, survival, death, inflammation and immune responses [20]. Endogenous ROS production during normal homeostasis contributes to mitogenic signaling; thus, decreasing the intracellular ROS levels is an attractive method for the inhibition of cancer growth. Conversely, excessive ROS, also called oxidative stress, may damage some organisms, resulting in cell death for both tumors and healthy cells. Interestingly, various anti-cancer drugs have been shown to activate ROS-mediated autophagy which in turn leads to cytoprotective regulation, induction of apoptosis induction or both.

Natural products show a broad spectrum of biological activities against a variety of diseases, including infections, immune system disorders, neurological disorders, and cancers [21]. Deoxypodophyllotoxin (DPT), isolated from *Anthriscus sylvestris*, is a naturally occurring flavolignan in medicinal herb plants. DPT has been shown to have potential anti-proliferative and anti-tumor activities in various cancer cell lines as well as anti-inflammatory and anti-viral activity [22]. Despite these findings, the details of the cellular and molecular mechanisms underlying the DPT-mediated cell death are poorly understood. Hence, the purpose of this present study was to investigate the molecular mechanisms in DPT-mediated apoptosis/autophagy pathway.

## RESULTS

### DPT inhibits cell viability and induces apoptosis in prostate cancer cells

**Figure 1:**
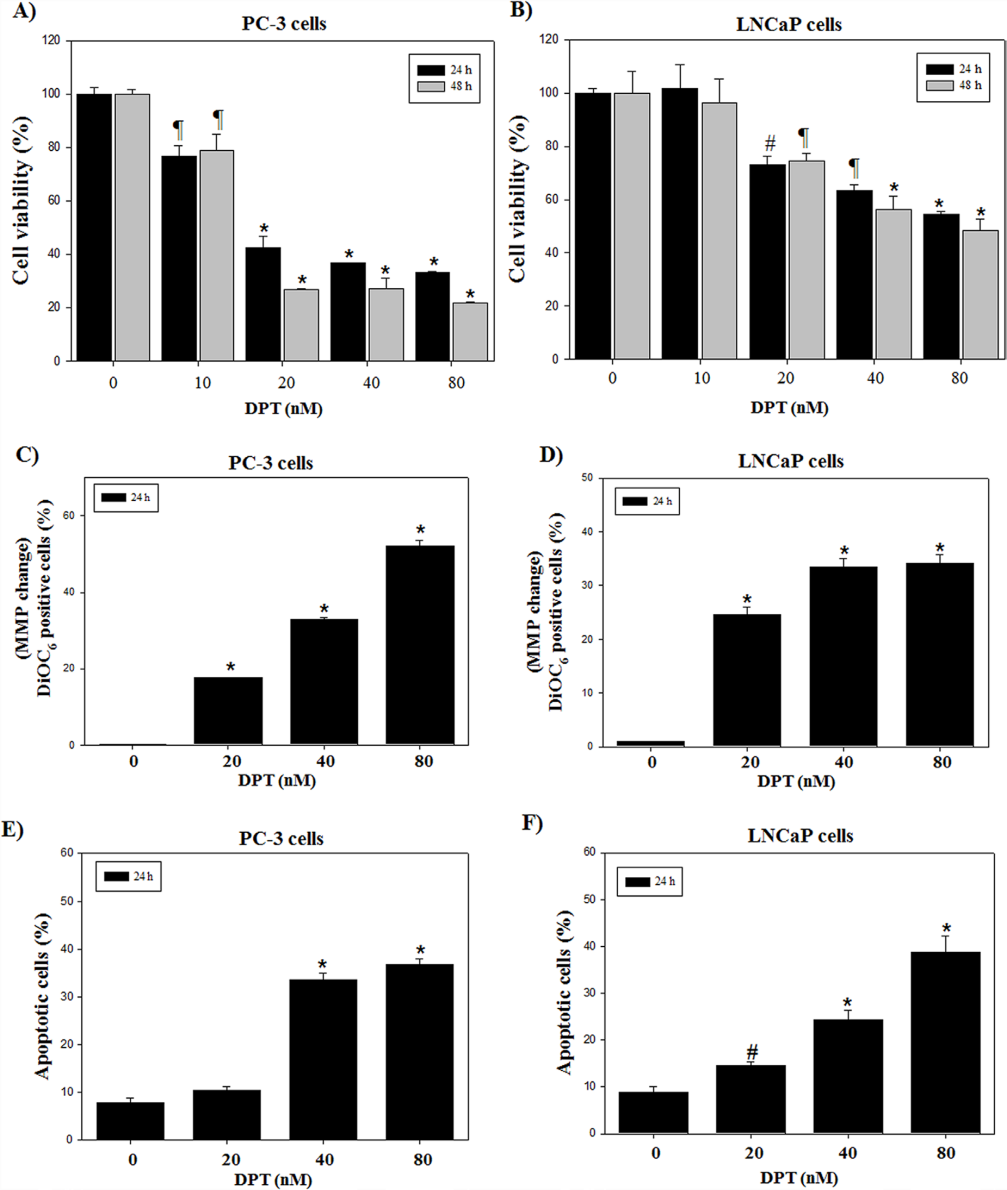
DPT inhibits cell viability and induces apoptosis in prostate cancer cells **(A, B)** Cell viability. **(C, D)** Changes of MMP. **(E, F)** Apoptosis analysis. The PC-3 (A, C, E) and LNCaP (B, D, F) cells were treated with various concentrations of DPT for 24 h. Cell viability was determined by MTT method as described in *Materials and Methods*. MMP was determined with a DiOC_6_ fluorescence dye by flow cytometry. Quantities of apoptosis were determined by annexin-V/propidium iodide (PI) staining. Data are presented as mean ± SD (n = 3 in each group). ^#^p < 0.05, ^¶^p < 0.01, ^*^p < 0.001 vs. the control group.

To investigate the cytotoxic effect of DPT, prostate cancer cells including PC-3 and LNCaP were treated with various concentrations of DPT for 24 and 48 h. DPT inhibited the cell viability of hormone-independent PC-3 cells and hormone-dependent LNCaP cells in a dose- and time-dependent manner with approximately 50% growth inhibition at a concentration of 20 and 40 nM for 24 h, respectively (Figure 1A and 1B). The PC-3 cells were more susceptible to DPT than the LNCaP cells. In contrast, normal prostate RWPE-1 cells were relatively more resistant to DPT than prostate cancer PC-3 and LNCaP cells (Supplementary Figure 1A). To summarize, DPT inhibited to a greater extent the cell viability of prostate cancer cell lines than that of normal prostate cells, showing a higher drug sensitivity to the prostate cancer cells. Mitochondrial dysfunction has been shown to participate in apoptotic cell death, resulting from the release of cytochrome c and activation of caspase-9 and -3, which are the key steps in apoptosis signaling. The effect of DPT on mitochondrial membrane potential (MMP) has been examined by using DiOC_6_, a fluorescent dye, in PC-3 and LNCaP cells. The result showed that significant polarization of MMP significantly occurred after the treatment with DPT in a dose-dependent manner (Figure 1C and 1D). Quantification of apoptosis was measured by annexin-V-FITC/PI staining. The positive cells of annexin-V-FITC staining were approximately 33.5% and 22.8% at 40 nM DPT for 24 h in PC-3 and LNCaP cells, respectively (Figure 1E and 1F). However, the percentage of apoptosis in RWPE-1 cells was 12.5% less than the induction of apoptosis in prostate cancer cells (Supplementary Figure 1B), indicating that DPT shows selective apoptosis in the prostate cancer cells. Additionally, an increase of Bax (pro-apoptotic factor)/Bcl-2 (anti-apoptotic factor) ratio, activation of caspase-3 and cleavage of PARP were detected in PC-3 and LNCaP cells (Supplementary Figure 1C and 1D). Taken together, the chemo-sensitivity of the prostate cancer PC-3 and LNCaP cells, especially PC-3, was higher than that of the normal prostate RWPE-1 cells, resulting from an induction of increased apoptosis. Thus, hormone-independent PC-3 cells were chosen to further investigate the mechanism associated with the DPT-induced cell death.

### DPT triggers ROS production from the mitochondrial compartment of PC-3 cells

**Figure 2:**
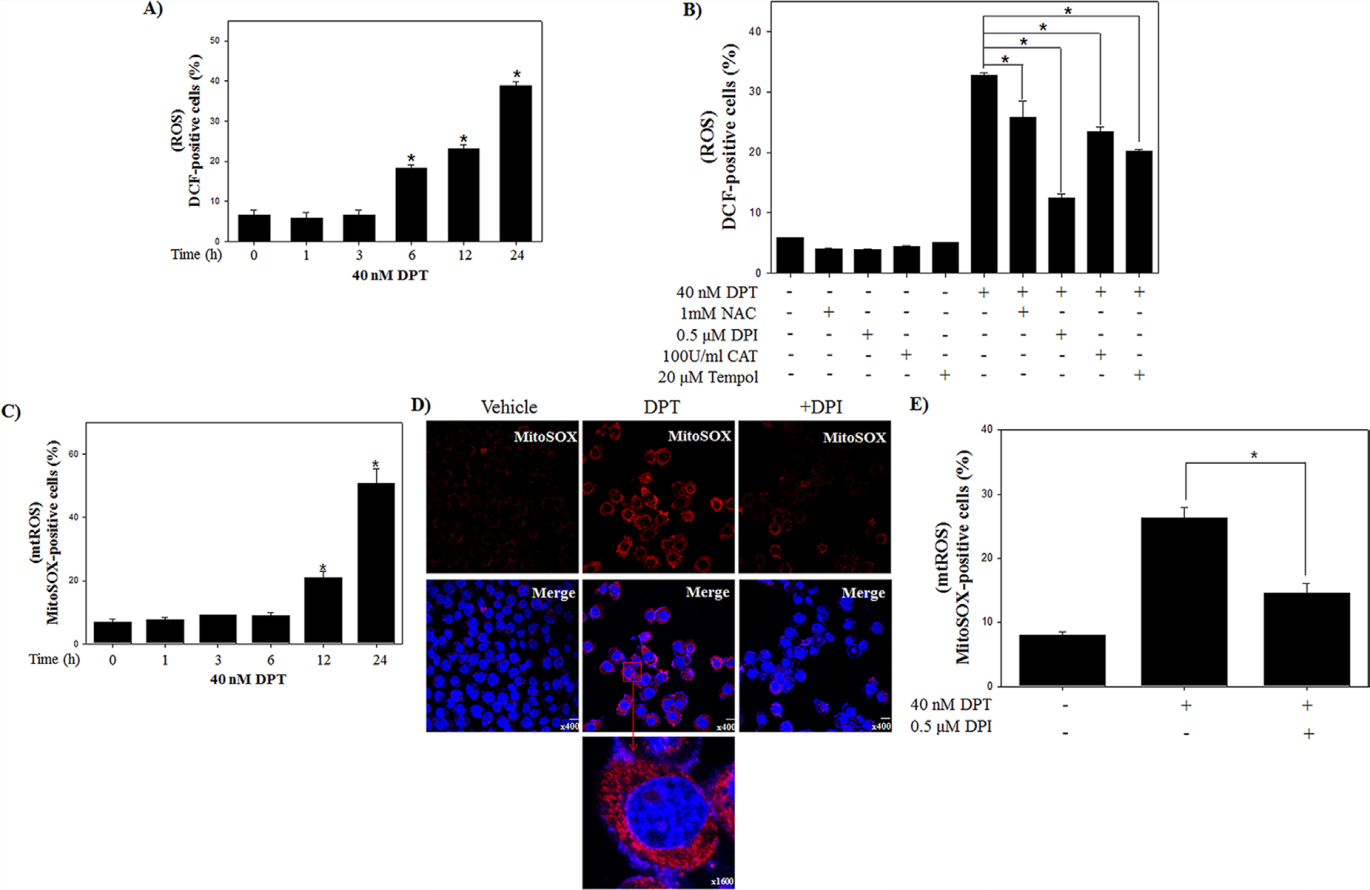
DPT triggers the generation of ROS in prostate cancer PC-3 cells **(A, B)** ROS production. The PC-3 cells were treated with 40 nM DPT for indicated time in the presence or absence of ROS inhibitors, such as NAC, DPI, CAT and Tempol. The ROS was determined with a fluorescence dye of DCFH-DA by flow cytometry. **(C)** The mitochondrial ROS. **(D)** Representative images of Mitochondrial ROS. **(E)** Quantification of mitochondrial ROS production. The PC-3 cells were treated with 40 nM DPT for 24 h in the presence or absence of DPI. Mitochondrial ROS were determined with a fluorescence of MitoSOX, a selective mitochondrial ROS dye, by flow cytometry. Representative images of Mitochondrial ROS were obtained by confocal fluorescence microscopy. After fixation and permeabilization, nuclei were counter-stained with DAPI. Scale bar, 20 μm. Quantification of mitochondrial ROS production was performed by flow cytometry. Data are presented as mean ± SD (n = 3 in each group). ^*^p < 0.001 vs. the control group.

**Figure 3:**
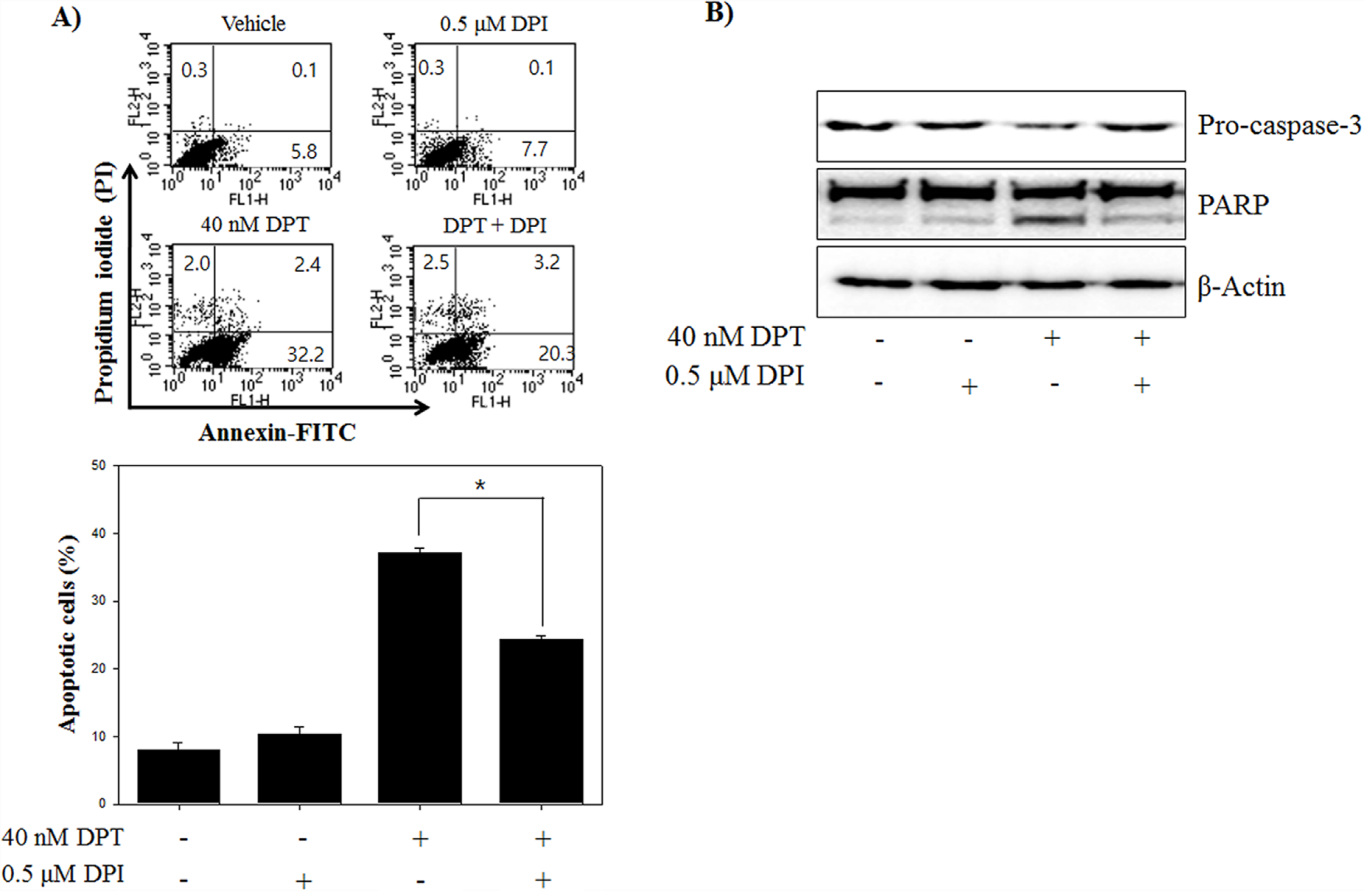
DPT induces the apoptosis through mitochondrial ROS in PC-3 cells **(A)** Apoptosis analysis. **(B)** Protein expression. The PC-3 cells were treated with 40 nM DPT for 24 h with the presence or absence of DPI. Apoptotic cells were analyzed by flow cytometry. Protein expression was analyzed by western blotting with antibodies for pro-caspase-3 and PARP. β-Actin was used as a loading control. Data are presented as mean ± SD (n = 3 in each group). ^*^p < 0.001 vs. the control group.

Several studies previously reported that intracellular ROS production is the byproduct of normal cellular oxidative processes involved in the initiation of apoptotic signaling [23]. We examined the effect of DPT on ROS production using flow cytometry with DCFH-DA, an indicator for ROS. As shown in Figure 2A, an intracellular ROS levels were significantly increased by the treatment of DPT in a time-dependent manner in PC-3 cells. To identify the dependency of DPT-induced apoptosis on ROS, PC-3 cells were treated with DPT in the presence or absence of various ROS inhibitors (general ROS scavenger, NAC and Tempol; mitochondrial ROS inhibitor, DPI; H_2_O_2_ scavenger, CAT). The results showed that DPT-triggered ROS production was recovered by a pre-treatment with the various ROS inhibitor. Interestingly, DPI significantly recovered DPT-triggered ROS production (Figure 2B). DPI has been reported to inhibit mitochondrial ROS production via the suppression of superoxide and H_2_O_2_ generated from mitochondrial respiration [24]. Because DPI has a strong inhibitory effect in DPT-triggered ROS, we investigated the mitochondrial ROS production using MitoSOX, a selective fluorescent dye of mitochondria-derived ROS. The result showed that DPT significantly induced mitochondrial ROS in a time-dependent manner (Figure 2C). Next, the visualization of ROS generated from mitochondria was examined by a confocal microscopy after MitoSOX staining. Here, confocal microscopy image showed that DPT enhanced the fluorescence intensity of MitoSOX in the mitochondrial portion and the fluorescence was decreased by DPI (Figure 2D). The aforementioned phenomenon was re-confirmed by a flow cytometry analysis (Figure 2E). These results suggested that DPT stimulates ROS production mainly generated from the mitochondria in PC-3 cells. Previous studies reported that ROS derived from the mitochondria are involved in apoptosis [25]. To identify whether the mitochondrial ROS production by DPT is associated with apoptotic cell death, we examined DPT-induced apoptosis in the presence or absence of DPI. As the results of pre-treatment with DPI, apoptotic cells were decreased as compared with DPT-treated cells (Figure 3A). In addition, pre-treatment with DPI provoked the expression of apoptosis-related proteins, resulting in increase of pro-caspase-3 and reduction of PARP cleavage (Figure 3B). These results indicated that mitochondrial ROS production plays an important role in the upstream pathway of DPT-induced apoptosis in PC-3 cells.

### DPT induces autophagic flux in PC-3 cells

**Figure 4:**
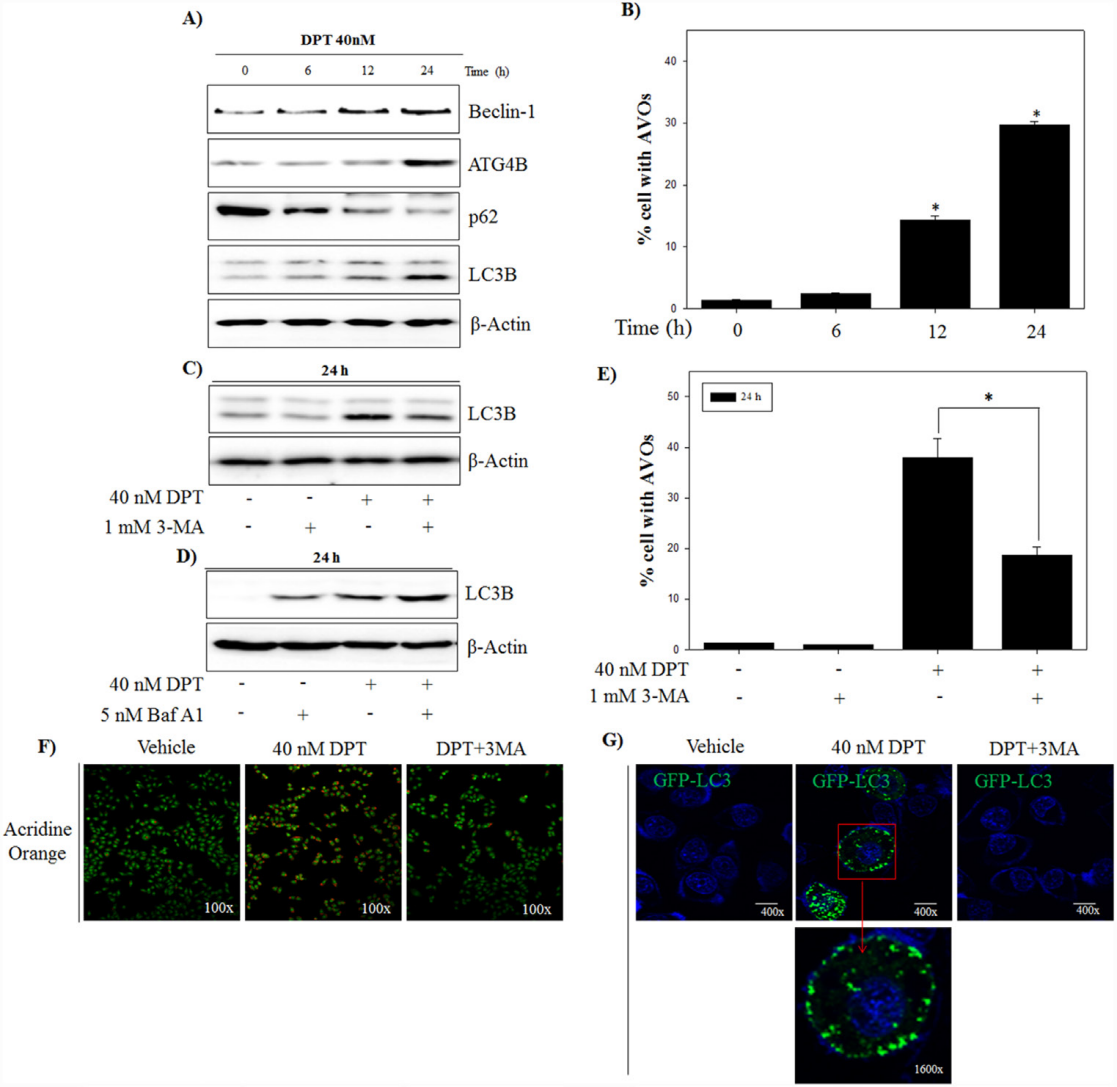
DPT induces autophagic flux in PC-3 cells **(A)** Protein expression. **(B)** AVO formation. The PC-3 cells were treated with 40 nM DPT for indicated times. Protein expression was analyzed by western blotting with antibodies for Beclin-1, ATG4, p62 and LC3B. β-Actin was used as a loading control. Quantification of AVO accumulation was determined by AO and calculated by FL-3 channel of flow cytometry. **(C, D)** Protein expression. **(E)** AVO formation. **(F)** Confocal microscope with AO. The PC-3 cells were treated with 40 nM DPT for 24 h in the presence or absence of 3-MA and Bafliomycin A1(Baf A1). Protein expression was analyzed by western blotting with antibody for LC3B. β-Actin was used as a loading control. AVO accumulation was determined by AO and calculated by FL-3 channel of flow cytometry. Formation of AVO was stained with AO and visualized by confocal microscope. **(G)** Confocal microscope with GFP-LC3. After transfection with GFP-LC-3 as described in *Materials and Methods*, PC-3 cells were treated with 40 nM DPT for 24 h in the presence or absence of 3-MA. Fluorescence staining for GFP-LC3 was observed by confocal microscope. Data are presented as mean ± SD (n = 3 in each group). ^*^p < 0.001 vs. the control group.

Autophagy plays a critical role in the degradation of cytoplasmic proteins. The functional relationship between apoptosis and autophagy is complex in cancer [26]. To investigate whether DPT induces the autophagy in PC-3 cells, western blotting was applied to examine the expression of autophagy-related proteins including Beclin-1, ATG4B, LC3B and p62. As shown in Figure 4A, DPT increased the expression of Beclin-1and ATG4B, and conversion of LC3B-I to LC3B-II in a time-dependent manner, which is one of the key steps in autophagy. Moreover, DPT reduced the expression of p62, an autophagy-specific marker delivered to the lysosome for degradation, indicating a reflection of increased autophagic degradation. Next, in order to quantify of acidic vesicular organelles (AVO), which is another evidences of autophagy, flow cytometry analysis was performed using acridine orange (AO) staining. As a result, DPT caused an accumulation of AVO with time, which is a specific exhibition/characteristic of autophagosome formation (Figure 4B). To further prove that autophagic activation is DPT-dependent pathway, PC-3 cells were treated with DPT after pre-treatment with 3-methyladenine (3-MA), a specific inhibitor of autophagy. The results showed that pre-treatment with 3-MA reduced the LC3-II expression and accumulation of AVO (Figure 4C and 4D).

In addition, we checked the effect of DPT-induced autophagy by Bafilomycin A1 (Baf A1), which is an inhibitor of vacuolar H+ATPase that is used to block late-phase autophagy. As shown in Figure 4E, we found that Baf A1 treatment significantly increased the expression of LC3-II, indicating that the degradation of LC3-II is blocked by inhibiting autophagosome-lysosome fusion. A confocal microscopic analysis showed the similar results with fluorescence intensity of AVO. As shown in Figure 4F, DPT promoted the production of red fluorescent vesicles in the cytoplasm, whereas only a few of fluorescent vesicles were visualized in the control group and combination group with 3-MA and DPT. To investigate other critical evidences of autophagy, a transfection was performed using GFP-LC3 vector. As a result, DPT enhanced punctate accumulation of GFP-LC3, which is another characteristic of autophagy, which was also blocked via a pre-treatment of 3-MA (Figure 4G). Taken together, DPT-induced autophagic flux was proved by examining the expression of autophagy-related proteins, accumulation of AVOs, and punctate GFP-LC3 in PC-3 cells.

### DPT induces autophagy through PI3K/AKT-independent manner

**Figure 5:**
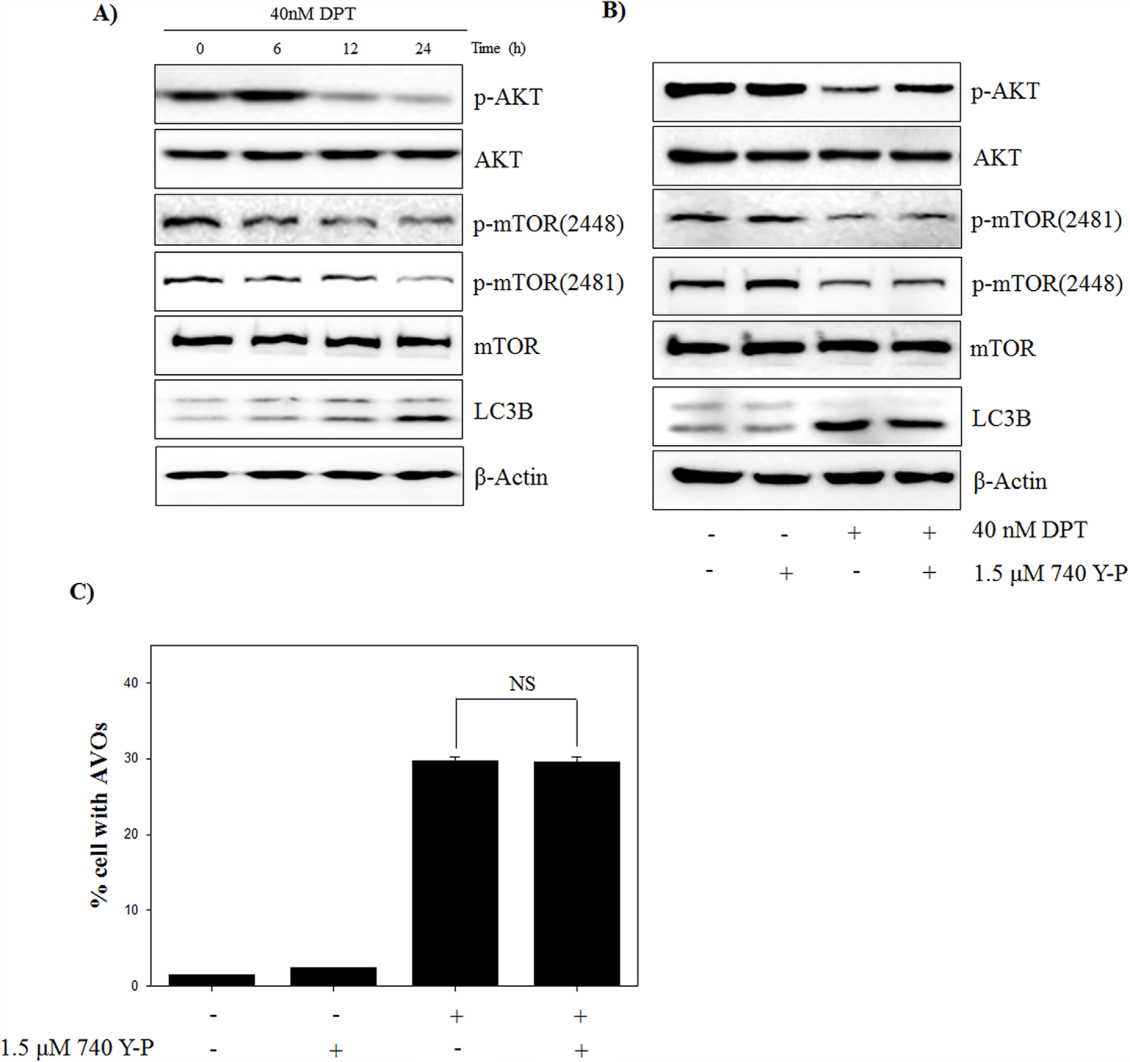
DPT inhibits PI3K/AKT/mTOR pathway of autophagy in PC-3 cells **(A, B)** Protein expression. **(C)** AVO formation. The PC-3 cells were treated with 40 nM DPT for indicated times in the presence or absence of 740 Y-P. Protein expression was analyzed by western blotting with antibodies for phospo-AKT, total AKT, phospo-mTOR at Ser 2448 and Ser 2481, total mTOR and LC3B. β-Actin was used as a loading control. Quantification of AVO accumulation was determined by AO and calculated by FL-3 channel of flow cytometry. Data are presented as mean ± SD (n = 3 in each group).

The kinase mammalian target of rapamycin (mTOR) is a major negative regulator of autophagy and a downstream target of the phosphatidylinositol 3 kinase (PI3K) and AKT pathways, which is activated by receptors of growth factors and stimulated cell growth, differentiation, and survival against apoptotic signals [27]. To examine whether PI3K/AKT/mTOR signaling is involved in DPT-induced autophagy, we investigated the expression of signaling-related molecules in PC-3 cells after DPT treatment. The results showed that DPT inhibited the phospho-AKT and mTOR levels at Ser 2448 and at Ser 2481 in PC-3 cells, but it had no effect on the total expression of AKT or mTOR (Figure 5A). To confirm the possible role of PI3K/AKT/mTOR pathways in DPT-induced autophagy, PC-3 cells were treated with DPT after activation of PI3K using the pharmacologic activator 740 Y-P. As a result, a pre-treatment with 740 Y-P recovered the inhibition of phospho-AKT expression, whereas it had no significant effect on phospho-mTOR expression (Figure 5B). Moreover, the LC3B expression, an autophagy marker protein, was not changed. Also, a pre-treatment with 740 Y-P also did not cause any changes in the accumulation of AVO induced by DPT (Figure 5C). These results suggested that DPT promotes the suppression of the PI3K/AKT/mTOR signaling pathway, which did not contribute to the activation of PI3K/AKT-dependent autophagy. Although the PI3K/AKT-dependent pathway plays a central role in the regulation of autophagy in mammalian cells, it is also involved in cell survival and inhibition of the apoptotic machinery in different cell types. To elucidate the involvement of PI3K/AKT pathway in the apoptotic process, apoptotic cells were analyzed after a pre-treatment with 740 Y-P. DPT-induced apoptosis was attenuated by a pre-treatment with 740 Y-P, as evidenced by increase of pro-caspase-3 and reduction of PARP cleavage (Supplementary Figure 2A and 2B). These results suggested that the inhibition of PI3K/AKT pathway by DPT leads to apoptosis rather than autophagy. Taken together, it seemed that DPT-induced autophagy is modulated in PI3K/AKT-independent manner on mTOR regulation.

### DPT promotes autophagy by activating ROS-induced ERK/MAPK signaling

**Figure 6:**
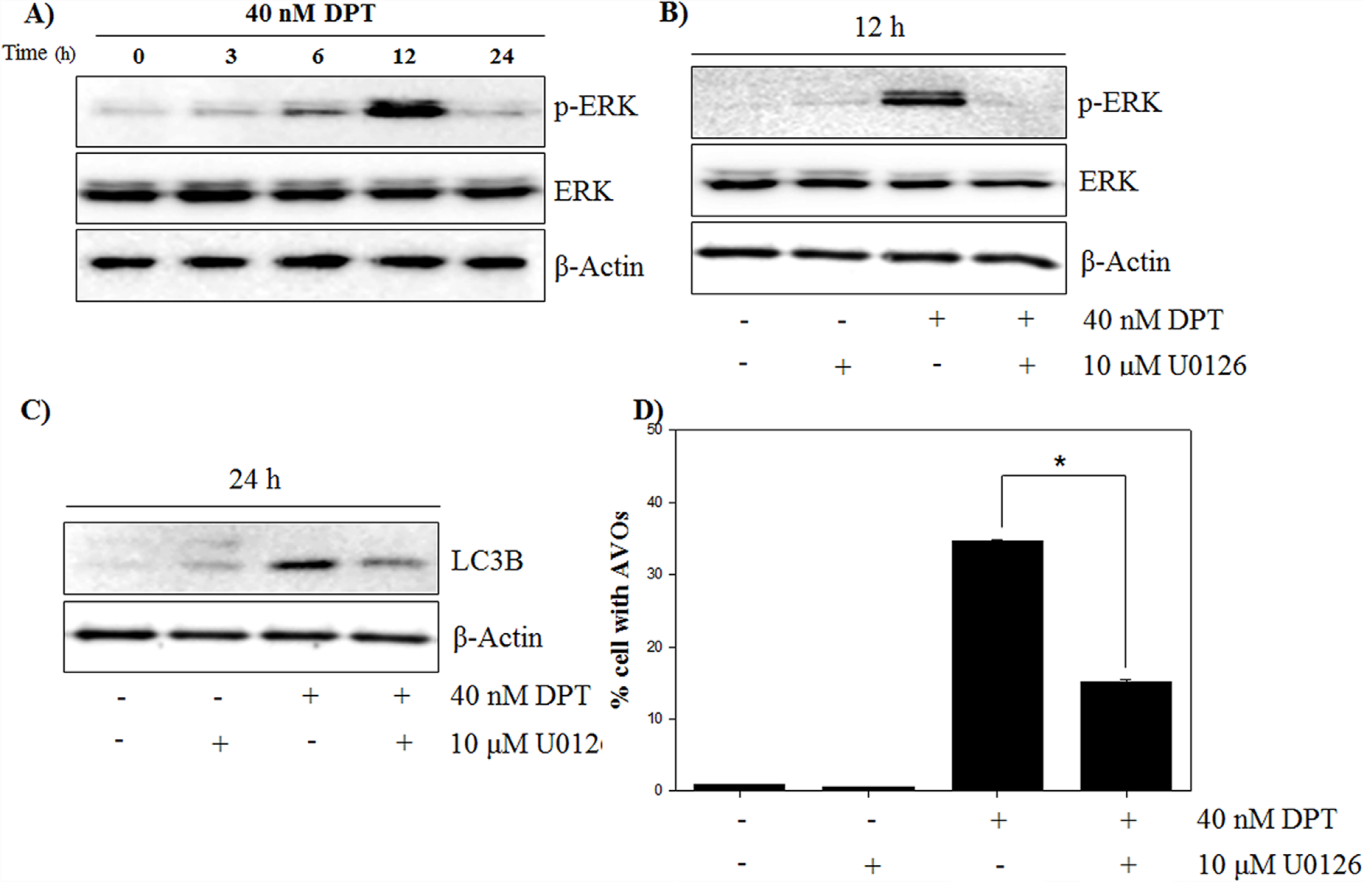
DPT promotes autophagy by activating ERK/MAPK signaling **(A, B, C)** Protein expression. **(D)** AVO formation. The PC-3 cells were treated with 40 nM DPT for indicated times in the presence or absence of U0126. Protein expression was analyzed by western blotting with antibodies for phospo-ERK and total ERK and for LC3B. β-Actin was used as a loading control. (D) Quantification of AVO accumulation was determined by AO and calculated by FL-3 channel of flow cytometry. Data are presented as mean ± SD (n = 3 in each group). ^*^p < 0.001 vs. the control group.

**Figure 7:**
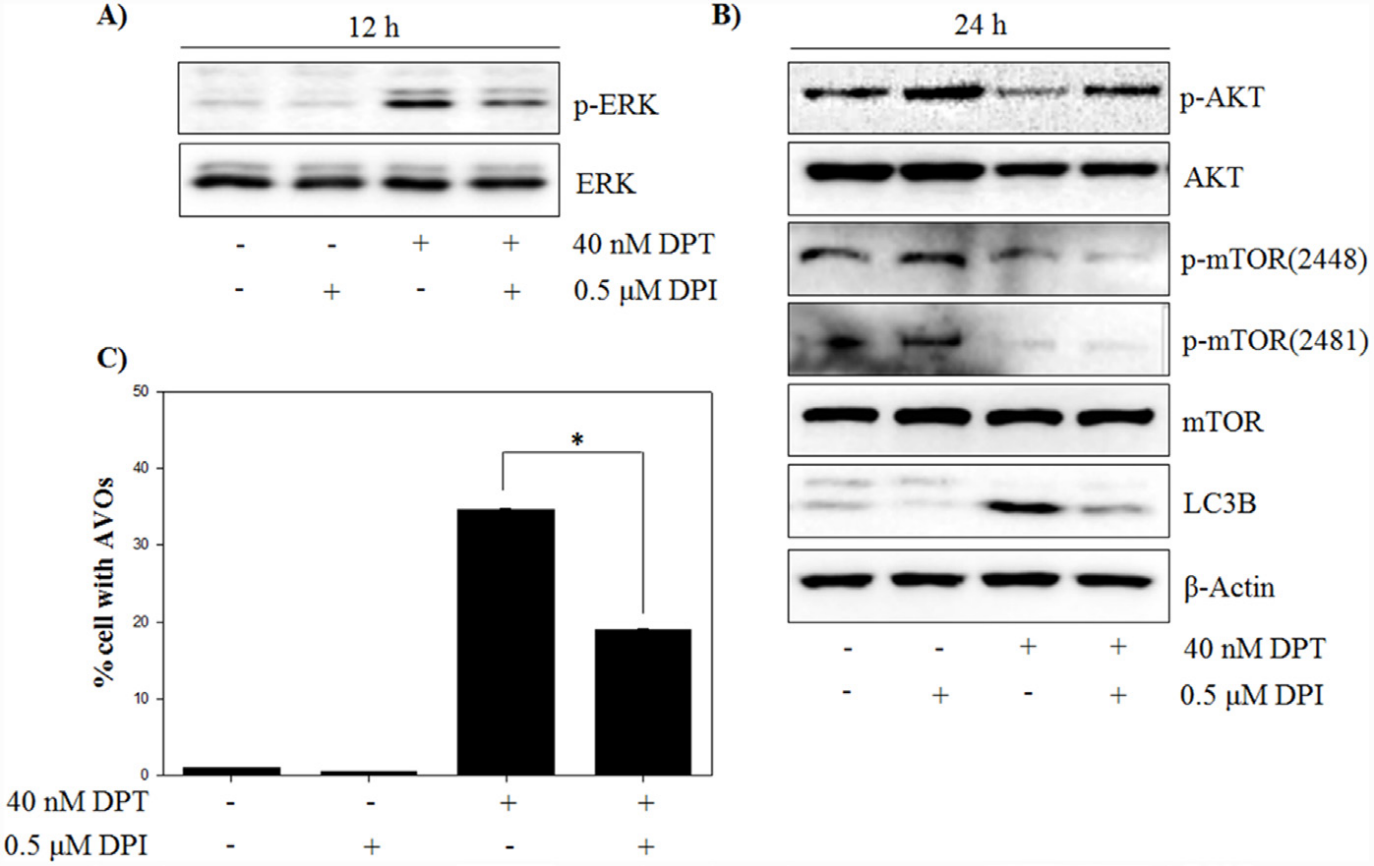
DPT induces ERK/autophagy signaling via mitochondrial ROS **(A, B)** Protein expression. **(C)** AVO formation. The PC-3 cells were treated with 40 nM DPT for indicated times in the presence or absence of U0126. Protein expression was analyzed by western blotting with antibodies for phospo-ERK and total ERK and for phospo-AKT, total AKT, phospo-mTOR at Ser 2448 and Ser 2481, total mTOR and LC3B. β-Actin was used as a loading control. Data are presented as mean ± SD (n = 3 in each group). ^*^p < 0.001 vs. the control group.

Many studies have demonstrated that autophagy is associated with MEK/ERK signaling pathway, which modulates gene expression, proliferation and metabolism [28]. To investigate whether the ERK signaling pathway is involved in the autophagy by DPT, at first, the expression of total ERK and phospho-ERK in response to DPT treatment was examined. As a result, phospho-ERK levels were significantly increased after DPT treatment at 12 h; but the total ERK levels were not changed (Figure 6A). It has been reported that the inhibition of ERK activity attenuates autophagy in hepatocellular carcinoma cells [29]. In this study, a pre-treatment with the MEK/ERK inhibitor U0126 resulted in a reduction of phospho-ERK level and a decrease of LC3B levels, consequently recovering DPT-induced autophagy (Figure 6B, 6C and 6D). These results showed that ERK activation is required for DPT-induced autophagy in PC-3 cells. ROS acts as a signaling molecule in many cellular processes, including growth, differentiation, apoptosis, and autophagy [30]. To investigate the functional link between ROS production and autophagy activation, the effect of DPI as an antioxidant was examined on the expression of ERK and autophagy activity. As expected, the activation of ERK by DPT was prevented by a pre-treatment of DPI (Figure 7A). Next, DPI significantly recovered the inhibition of phospho-AKT level and the induction of LC3B expression, although the mTOR-related proteins were not regulated (Figure 7B). In addition, the attenuation of ROS by DPI significantly decreased the number of AVO after DPT treatment (Figure 7C). Taken together, these data suggested that ROS production after DPT treatment promotes ERK activity and suppresses AKT activity, resulting in sequential autophagy in an mTOR-independent manner.

### Activated ERK-autophagy pathway plays a cell protective role in DPT-induced apoptosis

**Figure 8:**
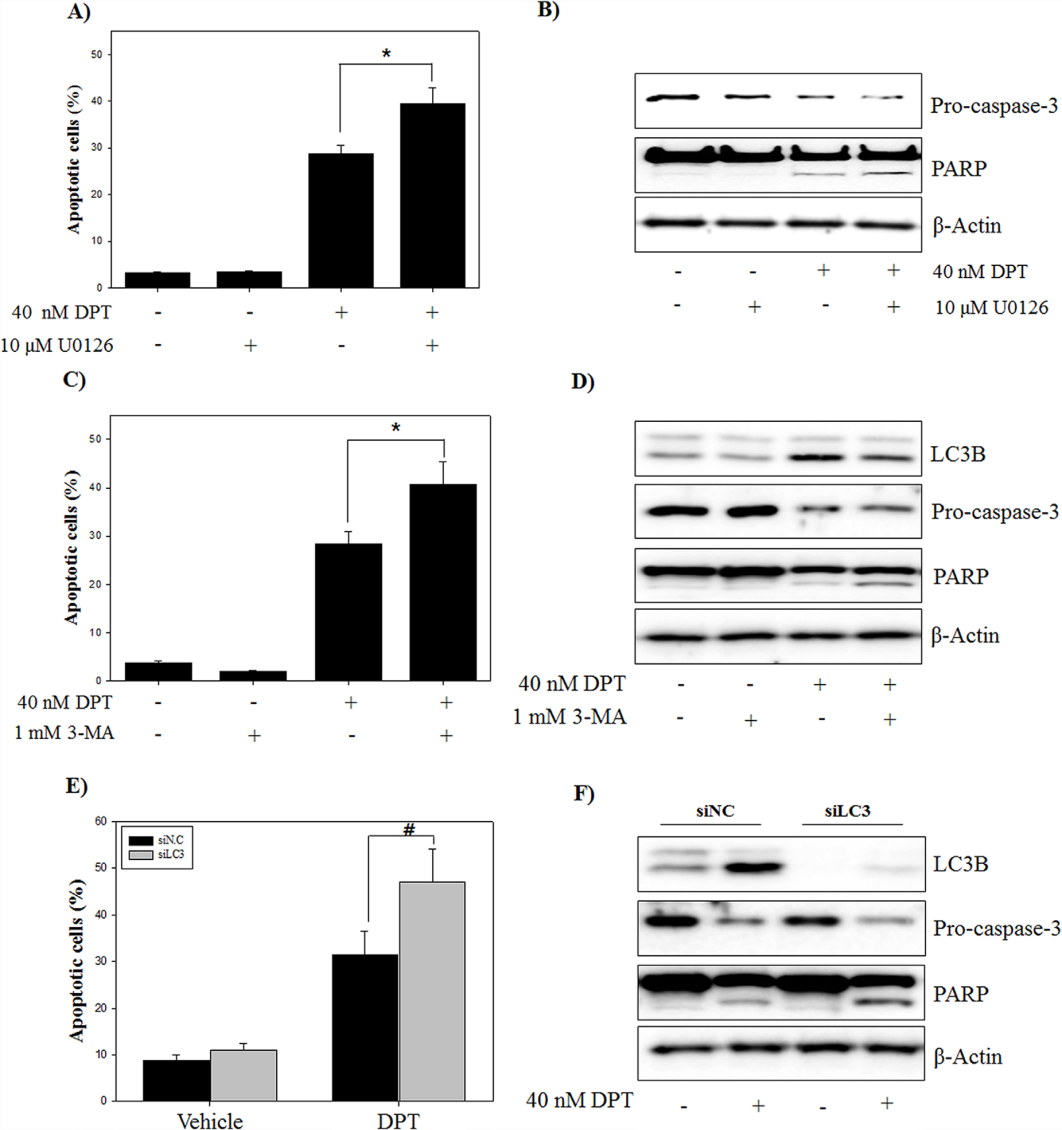
Inhibition of ERK/autophagy enhances DPT-induced apoptosis in PC-3 cells **(A)** Apoptosis analysis. **(B)** Protein expression. The PC-3 cells were treated with 40 nM DPT for 24 h in the presence or absence of U0126. Apoptotic cells were analyzed by flow cytometry. Protein expression was analyzed by western blotting with antibodies for pro-caspase-3 and PARP. β-Actin was used as a loading control. **(C)** Apoptosis analysis. **(D)** Protein expression. The PC-3 cells were treated with 40 nM DPT for 24 h in the presence or absence of 3-MA. Apoptotic cells were analyzed by flow cytometry. Protein expression was analyzed by western blotting with antibodies for LC3B, pro-caspase-3 and PARP. β-Actin was used as a loading control. **(E)** Apoptosis analysis. **(F)** Protein expression. The PC-3 cells transiently transfected with negative control siRNA, LC3B siRNA that were treated with 40 nM of DPT for 24 h. Apoptotic cells were analyzed by flow cytometry. Protein expression was analyzed by western blotting with antibodies for LC3B, pro-caspase-3 and PARP. β-Actin was used as a loading control. Data are presented as mean ± SD (n = 3 in each group). ^#^p < 0.01, ^*^p < 0.001 vs. the control group.

In previous reports, it has been shown that ERK-autophagy signaling plays as a protective role against drug-induced cell death [31]. To test the potential role of ERK signaling in DPT-induced apoptosis, apoptotic cells were analyzed after a pre-treatment with U0126, an ERK inhibitor. The results showed that DPT-induced apoptosis was significantly enhanced by a pre-treatment with U0126 (Figure 8A), confirming by recovered pro-caspase-3 reduction and PARP cleavage (Figure 8B). Next, to address the role of DPT-induced autophagy against apoptosis, two strategies were applied to inhibit DPT-induced autophagy: Pharmacologic (small molecule inhibitor, 3-MA) and genetic approaches (siRNA knockdown, LC3B). A pre-treatment with 3-MA markedly enhanced the DPT-induced apoptosis in PC-3 cells (Figure 8C). Reduction of LC3B and pro-caspase-3 expression and accumulation of cleaved PARP were also detected in DPT-treated cells (Figure 8D). Consistent with the pharmacological inhibition of autophagy, a knockdown of LC3B promoted DPT-induced apoptosis, as evidenced by regulation of proteins expression such as LC3B, pro-capase-3, and PARP (Figure 8E and 8F). These results suggested that the activation of ERK-autophagy plays a cytoprotective role in DPT-induced apoptosis in PC-3 cells.

### DPT inhibits tumor growth *in vivo* in PC-3 cells xenograft model

**Figure 9:**
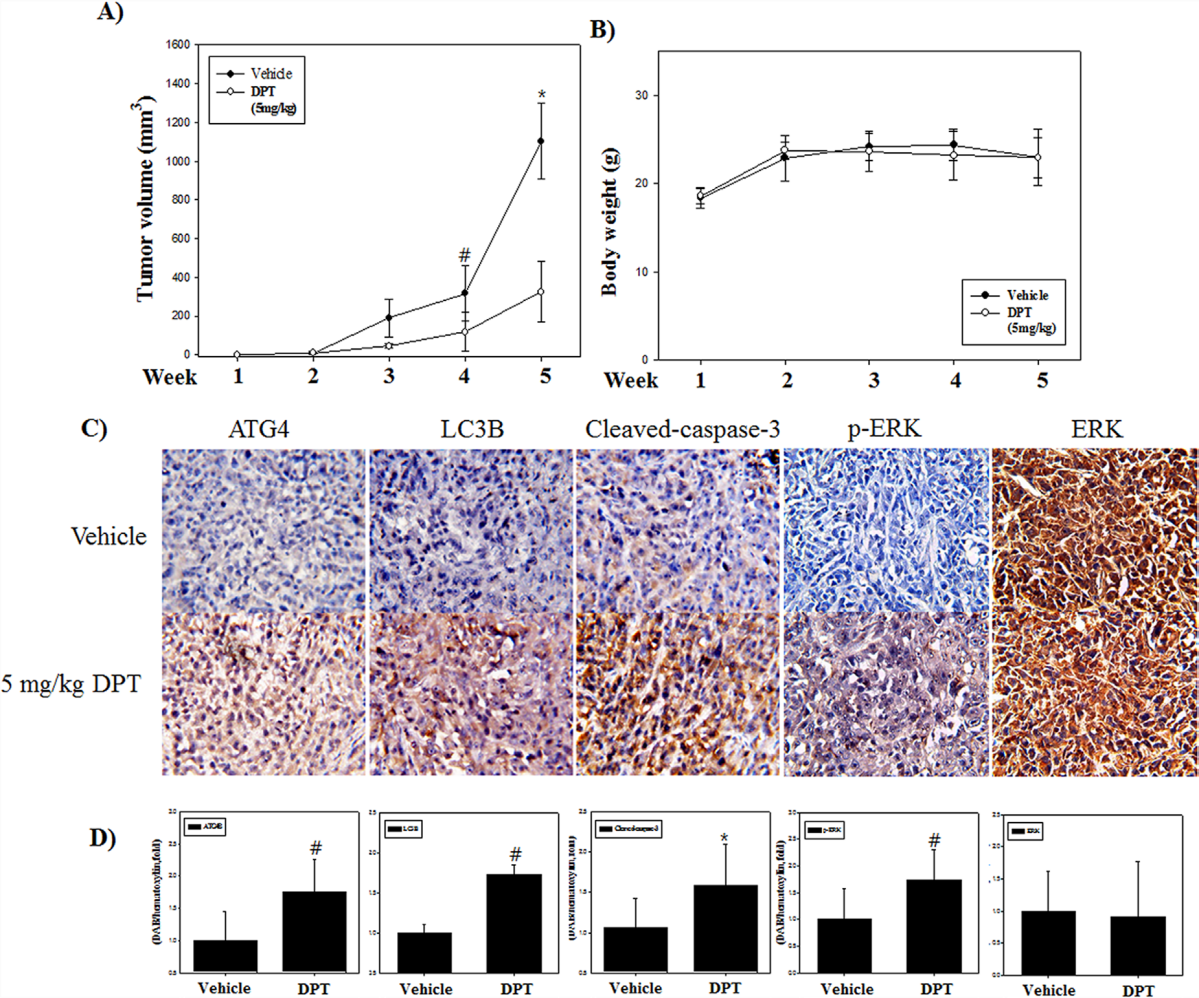
DPT inhibits growth and induces both apoptosis and autophagy of human prostate cancer xenograft *in vivo* **(A)** Tumor volume. **(B)** Mouse body weight. **(C)** Protein expression by IHC. **(D)** Quantification of protein expression. The PC-3 cells were inoculated subcutaneously in the right flank of BALB/c-nu mice. Mice received a daily oral administration of 5 mg/kg DPT for 5 weeks. Mice were sacrificed after the indicated treatments, and tumor volume and mouse body weight were measured. The expression levels of ATG4B, LC3B, cleaved caspase-3, phospho-ERK and total-ERK were examined by IHC. The average percentages of ATG4B, LC3B, cleaved caspase 3, phospho-ERK and total-ERK positive cells were quantified by HistoQuest software at x400 magnification in 5 randomly selected areas in each tumor sample. Data are presented as mean ± SD (n = 3 in each group). ^#^p < 0.05, ^*^p < 0.001 vs. the control group.

To extend our findings *in vitro*, we investigated the xenograft studies using PC-3 cells. Male BALB/c nude mice were randomly divided into 2 groups (vehicle and 5 mg/kg DPT) after a subcutaneously injection of PC-3 cells in the flank region. Mice were sacrificed after DPT treatment for 5 weeks and tumor tissues were collected. The results showed that tumor growth was delayed in the DPT treatment group compared with the vehicle group (Figure 9A). Meanwhile there was no difference in body weight change between the DPT treatment group and the vehicle group (Figure 9B). These results demonstrate an anti-tumor effect of DPT against prostate cancer a xenograft *in vivo* mouse model without any apparent signs of side effects. To further emphasize the clinical relevance for these findings, immunohistochemistry (IHC) analysis for ATG4B, LC3B, cleaved caspase-3, and phospho-ERK was carried out. Similar to our *in vitro* findings, the expressions of autophagy-related and apoptosis-related proteins were increased in the DPT treatment group. Moreover, the expression of phospho-ERK was increased without any changes of the total ERK expression, which was quantified by HistoQuest software (Figure 9C and 9D). Overall, these data demonstrated that DPT is highly effective against prostate cancer without toxic side effects, and the molecular mechanisms in response to DPT treatment were similar in both *in vitro* and *in vivo* systems.

## DISCUSSION

DPT, an analogue of podophyllotoxin, is a naturally occurring microtubule-depolymerizing flavolignan [32]. It has been reported that DPT displays anti-proliferative, anti-tumor, anti-inflammatory, and anti-viral activities [22, 33]. Many studies reported that DPT induces cell cycle arrest at the G2/M phase by inhibiting tubulin polymerization. However, to date, its precise mechanism in apoptosis and autophagy has been fully elucidated. In the present study, the mechanism of cell death by DPT was investigated in prostate cancer cells. Consistent with the previous findings [34], we found that DPT significantly inhibited the cell viability of prostate cancer cells, PC-3 and LNCaP. This was confirmed by an analysis of apoptosis-related proteins and MMP. Meanwhile, normal prostate RWPE-1 cells were significantly more resistant to growth inhibition by DPT than prostate cancer cells.

An induction of oxidative stress is expected to provide a powerful therapeutic modality in various cancers. In fact, many anti-cancer drugs and naturally occurring compounds have reported to have an anti-tumor effects via ROS-dependent apoptotic cell death in various cancer cells [35]. Identification of cellular sources of ROS production are critically important for ROS-mediated cell death in cancer. However, the origins of intracellular ROS are presently diverse, produced by various organelles (mitochondria, endoplasmic reticulum, peroxisomes) and enzymes (lipoxygenase, xanthine oxidase, cyclooxygenase, cytochrome P450 monooxygenase, nitric oxide synthase and NADPH oxidase). To investigate the probable sources of intracellular ROS production, ROS stimulated PC-3 cells were pre-treated with many ROS inhibitors in this study. Interestingly, general ROS scavengers (NAC and Tempol) and H_2_O_2_ scavenger (CAT) showed weak effects on ROS production, while mitochondrial ROS inhibitor (DPI) significantly reduced DPT-induced ROS production. This indicates that the mitochondrion is the primary cellular source of ROS induced by DPT in PC-3 cells. These findings were further confirmed by using MitoSOX, which is effectively used to detect mitochondrial-mediated superoxides in various cell types [36]. Although cancer cells generate a variety of endogenous ROS for survival, they are particularly sensitive to increased and prolonged ROS. It was reported that mitochondrial ROS production leads to the process of ROS-dependent cell death, as working in an upstream pathway in apoptosis [37]. Our results showed that DPI inhibits DPT-induced apoptosis through the regulation of apoptosis-related proteins, where mitochondrial ROS play an important role in the apoptotic process. Recently, it has been revealed that excess ROS produced from the mitochondria modulates the autophagy process [38]. Autophagy is an intracellular bulk degradation system and it is stimulated in response to external stressor to maintain cellular metabolism and cell survival. In addition, the functional relationship between apoptosis and autophagy is complex in cancer [25]. In this study, autophagy is induced by DPT via the up-regulation of Beclin-1, ATG4B, LC3B and p62, which are markers of autophagy, and finally via the accumulation of AVO in PC-3 cells. Moreover, a pre-treatment with 3-MA inhibited DPT-induced autophagy, resulting from a reduction of AVO accumulation and GFP-LC3 puncta formation. It has been reported that autophagy is associated with the PI3K/AKT/mTOR signaling pathway, which is a negative regulator of autophagy [39]. Although PI3K/AKT-dependent pathway is a major signaling regulating mTOR, PI3K/AKT-independent mTOR pathway also has also recently been reported [12]. Our results showed that DPT suppressed the levels of phospho-AKT and phospho-mTOR, suggesting that autophagy induction may be associated with the inhibition of the PI3K/AKT/mTOR signaling pathway. However, 740 Y-P, a pharmacologic activator of PI3K, had no significant effects on phospho-mTOR, LC3B expression and AVO accumulation. These results suggest that an autophagy induction by DPT was activated in a manner independent from the PI3K/AKT/mTOR signaling pathway in PC-3 cells. In cell homeostasis, AKT is activated by its phosphorylation and induces cell survival through the regulation of gene expression with anti-apoptotic activity [40]. PI3K activator, 740 Y-P, attenuated DPT-induced apoptosis to regulate caspase-3 and PARP, suggesting that PI3K/AKT activation plays a role in the cell survival of DPT-treated PC-3 cells. Mitochondrial ROS are not only known as an apoptosis initiator, but also reported as an autophagy inducer [20, 41]. However, in this study, mitochondrial ROS suppression had no effect on phospho-mTOR expression, whereas it significantly restored the reduction of phospho-AKT and the induction of phospho-ERK, LC3B expression and AVO accumulation. This indicates that mitochondrial ROS induces mTOR-independent autophagy as well as apoptosis in PC-3 cells. As aforementioned, apoptosis and autophagy are closely interrelated with each other [2]. Interestingly, autophagy induction has been shown to promote the cancer cell survival, thereby counteracting or limiting the apoptotic effect of chemotherapeutic agents [42]. It has been reported that autophagy inhibits the apoptotic process by suppressing the release of pro-apoptotic factor, such as cytochrome c, from the mitochondria and activating the caspase cascade in cancer cells [43]. Inhibition of autophagy with 3-MA or LC3 knockdown enhanced the DPT-apoptosis through the up-regulation of apoptosis-related protein, suggesting that autophagy acts as a cytoprotective role in prostate cancer cells. It has been well established that the MAPK signaling pathway is pivotal for signal transduction to regulate critical cellular responses such as proliferation, apoptosis, autophagy, differentiation and senescence [44]. Among the MAPK pathways, the activation of ERK signaling has been involved in autophagy process for several stresses, including amino acid deprivation and anti-cancer agents [15]. In this study, the pharmacological inhibition of ERK signaling attenuated DPT-induced autophagy and significantly increased its apoptosis in PC-3 cells. Thus, it seems that ERK activated by DPT, induces autophagy, exerting a protective role against apoptosis in PC-3 cells.

Furthermore, apoptosis and autophagy induced by DPT in prostate cancer PC-3 cells *in vitro* were additionally reconstituted in the experimental xenograft mouse model with *in vivo* PC-3 cells. *In vivo* studies showed that DPT inhibited tumor growth without any changes to the body weight, suggesting that DPT treatment did not exhibit toxic side effects. Immunohistochemistry (IHC) analysis demonstrated that DPT induced the expression of ATG4, LC3B and phospho-ERK, indicating the induction of cell survival pathways, as well as the expression of the cleaved caspase-3. We recommend that DPT might be a potential anti-cancer drug combined with autophagy inhibitors for patients with prostate cancer, through the regulation of apoptosis and autophagy via ROS generation.

## MATERIALS AND METHODS

### Materials

3-(4,5-Dimethyl-thiazol-2-yl)-2,5 diphenyltertrazolium bromide (MTT), propidium iodide (PI), 6-diamidino-2-phenylindole dihydrochloride (DAPI), 3-methyladenine (3-MA), Bafilomycin A1(Baf A1), acridine orange (AO), 2',7'-dichlorfluorescein-diacetate (DCFH-DA), N-acetyl-L-cysteine (NAC), and catalase (CAT) were purchased from Sigma Chemical Co. (St. Louis, MO, USA). Diphenyleneiodonium (DPI), MnTBAP and U0126 were purchased from Calbiochem (Merck, Darmstadt, Germany). Tempol and 740 Y-P were purchased from TOCRIS (Bristol, UK). FITC Annexin-V Apoptosis Detection kit was purchased from BD Bioscience (San Jose, CA, USA). The ECL Western Kit was purchased from iNtRON Biotechnology (Seongnam, South Korea). Beclin-1, ATG4B, LC3B, phospho-mTOR, total-mTOR, β-actin, phospho-AKT, total AKT, phospho-ERK, total ERK, phospho-p38, total p38, phospho-JNK, total JNK, Bcl-2, pro-caspase-3, cleaved caspase-3 and PARP were purchased from Cell Signaling Technology (Beverly, MS, USA) and Santa Cruz Biotechnology (Santa Cruz, CA, USA), respectively. The goat-anti-mouse IgG and goat-anti-rabbit secondary antibody were purchased from Enzo Life Science (Farmingdale, NY, USA). MitoSOX was purchased from Invitrogen (Grand Island, NY, USA). Deoxypodophyllotoxin (DPT) was isolated from *Anthriscus sylvestris* roots and its structural identity was determined by nuclear magnetic resonance analysis as described previously [45]. The compound was confirmed to be >95% pure by high performance liquid chromatography.

### Cell lines and cell culture

Human prostate cells lines, PC-3, LNCaP and RWPE-1, were obtained from the American Type Culture Collection (ATCC, Manassas, VA, USA). Prostate cancer PC-3 cells and LNCaP cells were cultured in Dulbecco’s modified Eagle’s minimal medium (DMEM, WelGENE, Daejon, South Korea) and Roswell Park Memorial Institute (RPMI) 1640 (WelGENE, South Korea) supplemented with 10% FBS and 1% penicillin-streptomycin solution with 5% CO_2_ at 37°C, respectively. Normal prostate epithelial RWPE-1 cells were cultured in keratinocyte serum-free media (K-SFM) containing 2.5 μg of epidermal growth factor (EGF), 25 mg of bovine pituitary extract (BPE, Invitrogen, Carlsbad, CA, USA) and 1% penicillin-streptomycin solution with 5% CO_2_ at 37°C.

### Cell viability assay

Cell viability was determined by using the MTT assay. Human prostate cancer PC-3 and LNCaP cells and human normal prostate RWPE-1 cells were seeded at a density of 1×10^4^ cells/well in a 48-well culture dish. The prostate cells were treated with DPT of various concentrations for 24 and 48 h. After incubation, cells were treated with 0.5 mg/ml of MTT solution for further 3 h incubation at 37°C and 5% CO_2_ atmosphere. The precipitates were dissolved in dimethyl sulfoxide (DMSO) to dissolve the MTT-fomazan complex. Colorimetric analysis was performed on a microplate reader (Molecular Devices, Sunnyvale, CA, USA) at a wavelength of 540 nm. The cell viability was determined by their relative percentage of the treated cells to the untreated cells by comparing their optical densities.

### Measurement of mitochondrial membrane potential (MMP, ΔΨ)

MMP was determined by using the fluorescent dye DiOC_6_. PC-3 and LNCaP cells were seeded at a density of 5×10^4^ cells/well in a 6-well culture dish and treated with different concentrations of DPT for 24 h. The cells were harvested and stained with 100 nM DiOC_6_ at 37°C for 30 min. Cells were then analyzed by flow cytometry (FACSCalibur, Becton Dickinson, San Jose, CA, USA) with CellQuest analysis software (Becton Dickinson).

### Annexin V/propidium iodide (PI) analysis

Quantitative analysis of apoptotic cells was performed by using FITC Annexin-V Apoptosis Detection kit. PC-3 and LNCaP cells were seeded at a density of 5×10^4^ cells/well in a 6-well culture dish and treated with different concentrations of DPT for 24 h. Then the cells were washed with phosphate buffered saline (PBS). Harvested cells were mixed in 1× binding buffer and stained with an annexin V/PI at room temperature for 15 min. Then, the stainied cells were detected by flow cytometry and analyzed by the CellQuest software.

### Measurement of intracellular reactive oxygen species (ROS) and mitochondrial ROS

Total intracellular ROS and mitochondrial ROS levels were measured by DCFH-DA and MitoSOX, respectively. Human prostate cancer PC-3 cells were cultured in a 6-well plates at a density of 5×10^4^ cells/well. After exposure to DPT for the indicated times, the cells were incubated with DCFH-DA or MitoSOX at a final concentration of 10 μM or 5 μM for 15 min at 37°C, respectively. Cells were washed twice with PBS and fluorescence positive cells were measured with flow cytometry and analyzed with CellQuest analysis software.

### Visualization of mitochondrial ROS

To visualize mitochondrial ROS, an immunofluorescence assay was performed. Briefly, the PC-3 cells were seeded on a cover-glass bottom dish and treated with 80 nM DPT for 24 h. Then the cells were incubated with 5 μM MitoSOX for 15 min at 37°C and fixed with 4% paraformaldehyde for 10 min at room temperature. After fixation, cells were subsequently permeabilized with 0.1% Triton X-100 for 10 min and blocked with 5% BSA. Then the cells were washed twice with PBS and then incubated with 1 μg/ml DAPI solution at 4°C for 15 min. Images were acquired using a confocal microscope (Olympus, Tokyo, Japan).

### Detection of acidic vesicular organelles (AVO)

To determine the AVO, acridine orange (AO) staining was used. The PC-3 cells were seeded at a density of 1×10^4^ cells/well in a 6-well culture dish and incubated for 24 h. After treatment with 80 nM DPT for indicated times, cells were stained with 1 μg/ml AO in serum-free medium for 15 min and then washed twice with PBS. To quantify the number of AVO, the stained cells were analyzed by FL-3 channel of flow cytometry and calculated using CellQuest software. Also, images of AVO formation were immediately visualized using a confocal microscope. The cytoplasm and nucleus of cells fluoresced bright green, whereas the acidic autophagic vacuoles exhibited bright red.

### Detection of GFP-LC3 puncta

The PC-3 cells were plated at a density of 1×10^4^ cells on a cover-glass bottom dish and incubated for 24 h. Transfection was carried out with Polyplus transfection reagent (Illkirch, France) according to the manufacturer’s protocol. PC-3 cells were transfected with 1 μg of GFP-LC3 plasmid DNA (kindly provided by Dr. Young-Kyo Seo, KRIBB, South Korea) for further 24 h incubation and then treated with 80 nM DPT for 24 h. After fixation, cells were subsequently permeabilized with 0.1% Triton X-100 for 10 min and blocked with 5% BSA. Then the cells were washed twice with PBS and then incubated with 1 μg/ml DAPI solution at 4°C for 15 min. The density of GFP-LC3 fluorescence was observed by a confocal microscope.

### RNA interference of LC3B

The PC-3 cells were transfected with short interfering RNA corresponding to LC3B (sense: 5’-GCACCUUCGAACAAAGAGUTT-3’ and antisense: 5’-CUCUUUGUU CGAAGGUGCTT-3’) and negative control siRNA (Cosmogenetech, Seoul, South Korea) using Polyplus transfection reagent, according to the manufacturer’s protocol. After incubation for 4 h, media was replaced with complete medium containing 10% FBS and antibiotics. The cells were then incubated for an additional 24 h and treated with DPT. Then cells were collected and cell lysates were subjected to western blotting of LC3B and PARP.

### *In vivo* tumor growth analysis

PC-3 cells (2×10^6^ cells) suspended in 100 μl PBS were inoculated subcutaneously in the right flank of 5-week-old BALB/C nude mice (Orient, Busan, South Korea). After tumor formation, divided randomly into two groups of 3 each, which consisted of vehicle (PBS) control and 5 mg/kg DPT treatment orally administered for every day. After 5 weeks of treatment, mice were sacrificed and tumor tissues were harvested for western blotting and immunohistochemistry (IHC) analysis. Tumor sizes were measured weekly to observe dynamic changes in tumor growth and calculated by a standard formula: volume= (length × width^2^)/2. The body weight of mice was measured weekly to evaluate the systemic toxicity of the drug. All animal experimental procedures were approved and monitored by Institutional Animal Care and Use Committee in Pusan National University (PNU-2015-0969).

### Immunohistochemical analysis and quantification

Tumor tissue specimens were fixed in 10% neutral buffered formalin and embedded in paraffin on slides after collection from the expired mice. The slides were deparaffinized in xylene, hydrated with graded alcohol, and treated with 3% H_2_O_2_ and 1% acetic acid in PBS for 15 min to block endogenous peroxidase activity. Antigen retrieval was completed in boiling sodium acetate buffer for 10 min. Then slides were incubated with 3% bovine serum albumin for 1 h, followed by incubation with ATG4B, LC3B, cleaved-capase-3, phospho-ERK and total ERK primary antibodies at 4°C overnight, respectively. After being washed five times with PBS, slides were incubated with horseradish peroxidase-conjugated secondary antibody in dark at 37°C for 1 h. Slides were visualized by 3,3’-diaminobenzidine (DAB, Sigma). Hematoxylin was used for background counterstaining. Quantification of immunoreactive image was measured by HistoQuest software (TissueGnostics, Vienna, Austria)

### Statistical analysis

Experiments were repeated at least 3 times with consistent results. Unless otherwise stated, data are expressed as the mean ± SD. ANOVA was used to compare the experimental groups to the control, whereas comparisons between multiple groups were performed using a Tukey’s multiple comparison test. The results were statistically significant at *p* < 0.05, *p* < 0.01, *p* < 0.001 *vs.* the untreated group.

## SUPPLEMENTARY MATERIALS FIGURES


