# Supplementary material for: Mitochondrial ROS activates ERK/autophagy pathway as a protected mechanism against deoxypodophyllotoxin-induced apoptosis
Source: Oncotarget. 2017 Dec 4;8(67):111581–96. doi: 10.18632/oncotarget.22875 (PMC5762344; doi:10.18632/oncotarget.22875)
Supplement: Supplementary file 1 [file oncotarget-08-111581-s001.pdf]

Mitochondrial ROS activates ERK/autophagy pathway as a protected mechanism against deoxypodophyllotoxin-induced apoptosis

SUPPLEMENTARY MATERIALS

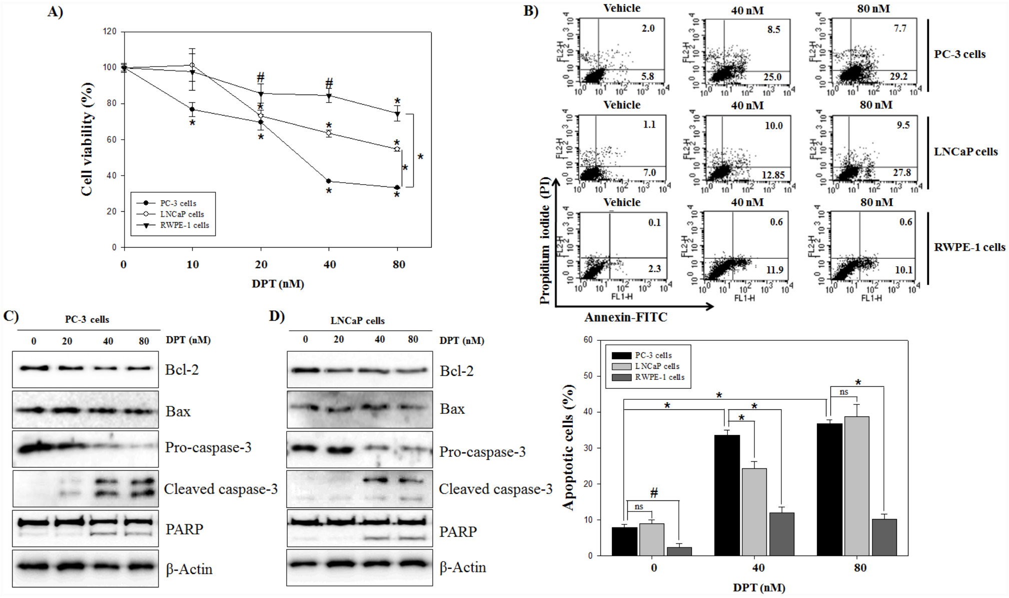

Supplementary Figure 1: DPT inhibits cell viability and induces apoptosis in cancer cell lines but not in normal cell lines. **(A)** Cell viability. Prostate cell lines were treated with with various concentrations of DPT for 24 h. Cell viability of prostate cell lines was determined by MTT method as described in *Materials and Methods*. **(B)** Apoptosis analysis of prostate cell lines was determined by annexin-V/PI staining. **(C, D)** Protein expression was analyzed by western blotting with antibodies for Bcl-2, Bax, pro-caspase-3, cleaved-caspase-3 and PARP. β-Actin was used as a loading control. Data are presented as mean ± SD (n = 3 in each group). #$p < 0.05$, $^{*}p < 0.001$ vs. the control group.

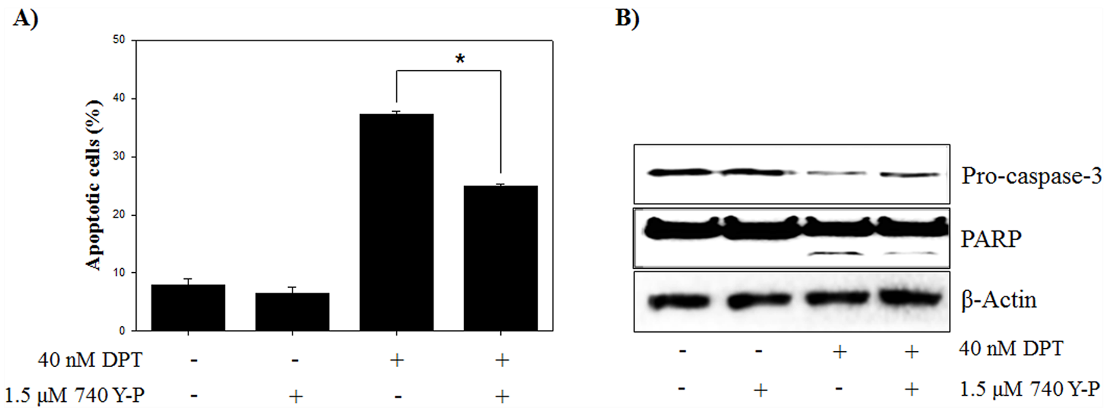

Supplementary Figure 2: PI3K/AKT signaling pathway is involved in promoting cell survival or blocking apoptosis in PC-3 cells. (A) Apoptosis analysis. **(B)** Protein expression. The PC-3 cells were treated with 40 nM DPT for 24 h with the presence or absence of 740 Y-P. Apoptotic cells were analyzed by flow cytometry. Protein expression was analyzed by western blotting with antibodies for pro-caspase-3 and PARP. β-Actin was used as a loading control. Data are presented as mean ± SD (n = 3 in each group). $^{*}p < 0.001$ vs. the control group.
